# The implementation of knowledge‐based planning with partial OAR contours for prostate radiotherapy

**DOI:** 10.1002/acm2.70004

**Published:** 2025-02-07

**Authors:** Ositomiwa O. Osipitan, David Wiant, Han Liu

**Affiliations:** ^1^ Department of Radiation Oncology Cone Health Cancer Center Greensboro North Carolina USA

**Keywords:** auto‐segmentation, knowledge‐based planning, partial contours

## Abstract

**Purpose:**

Intra‐ and inter‐observer contour uncertainty is a continuous challenge in treatment planning for radiotherapy. Our proposed solution to address this challenge is the use of partial contours for treatment planning, focusing on uninvolved or non‐overlapping portions of the organs‐at‐risk (OARs) with the planning target volume (PTV).

**Methods:**

The partial contours systematically eliminate overlapping regions. The partial contours were evaluated against fully contoured OARs. We incorporated advanced tools like knowledge‐based planning (KBP) to create treatment plans and artificial intelligence (AI) to create auto‐segmented contours. We developed two models, Rapid Plan (RP) and Rapid Plan partial uninvolved (RP_Part_Un), using 70 previous clinically approved volumetric arc therapy (VMAT) plans each prescribed with 70 Gy/28 fractions. From these models, we created three plans, RP, RP_Part_Un, and MIM AI_Part_Un. In this retrospective study, 60 prostate patients were analyzed using the three plans. For determining OAR sparing, *D*_max_ and *D*_mean_ along the percent volume receiving a dose over a range (V_10_ Gy V_70_ Gy) between each plan were compared. Geometric evaluations, dice similarity coefficient (DSC), and overlay index (OI) between the OAR contours from partial‐contoured manual structure sets and partial‐contoured AI structure sets were analyzed.

**Results:**

When comparing the *DSC* and *OI* for full contours to the partial contours, in both groups, all comparisons were significantly increased for both organs. This indicated the partial contours had a higher degree of concordance. In patients with SpaceOAR, RP_Part_Un plans exhibited significantly reduced bladder *D*_max_ and *D*_mean_ compared to RP plans, while rectum *D*_max_ and D_mean_ showed no significant differences. For patients without SpaceOAR, RP_Part_Un significantly lowered rectum *D*_mean_. MIM AI_Part_Un plans demonstrated lower rectum *D*_max_ in both patient groups.

**Conclusions:**

Partial contours, defined at a specified distance from the PTV, yielded dosimetry comparable to fully contoured plans, highlighting their potential efficacy in treatment planning.

## 1 INTRODUCTION

Prostate cancer is the second most common form of cancer in men. Its incidence rate is continuously increasing among men, and contributing factors are increasing age population, ethnicity, and heredity.^1^, ^2^, ^3^, ^4^ Despite the increasing numbers, the mortality rates are low and decreasing.^1^, ^2^, ^3^, ^4^ Advancements in health care have allowed for better treatment over time. However, there are still some challenges. One of which, in the field of radiation therapy (RT), is the accuracy and efficiency of delineating anatomical structures, such as the target and organs‐at‐risk (OARs) during treatment planning. The challenge is to achieve precise delineation to ensure that radiation is delivered to the tumor while minimizing exposure to healthy tissues. Many factors can affect this success, including expertise, medical imaging interpretation, the understanding of human anatomy, knowledge of tumor characteristics, and individual anatomy variations. Inaccurate contouring could potentially lead to a reduction of the dose delivered to the tumor site, lower local control, and increased dose to OARs.

In radiation treatments, time to treat and contouring consistency are important aspects of the treatment process. From retrospective observational studies when a patient's RT treatment is delayed from first diagnosis to when treatment begins, local tumor recurrence is indirectly affected.^5^ When planning, accurate contouring of the OARs can be a time‐consuming component and can have large inter‐ and intra‐observer variabilities. For prostate cancer patients, the bladder and rectum are OARs that are typically of concern due to their close proximity to the prostate. Additionally, physiological filling or gas may contribute to differing sizes among individuals that lead to variations in delineation even by expert observers.^6^ Incorrect, inadequate, or over‐contouring of these OARs is a major source of error in RT delivery and can lead to poor normal tissue sparing and increased toxic effects.

Incorporating advanced tools like knowledge‐based planning (KBP) and artificial intelligence (AI) represents a pivotal step toward ameliorating issues‐concerning contouring consistency, and ensuring uniformity in contouring methodologies. KBP is based on a statistical model that estimates dose‐volume histograms (DVHs) and generates dose‐volume objectives that can be used for photon optimization from a library of clinically accepted plans with high quality. The benefits of using this technique, which include the reduction in plan quality variance and planning time, have been described previously.^7^, ^8^, ^9^, ^10^ Prostate is a highly investigated disease site using KBP methods.^11^, ^12^ Many have highlighted that KBP can create comparable or at times better dosimetric results than manually optimized plans.^7^, ^8^, ^9^, ^10^, ^11^, ^12^

The prominence of AI is steadily escalating within radiation oncology, responding to the evolving demands of a technically challenging field like radiotherapy. One area in which AI is implemented is contouring. These auto‐contouring applications could revolutionize radiotherapy treatments. Delineating radiotherapy target volumes and OARs is a key step in the treatment process. However, it is a very strenuous step that produces variability and can take a long time depending on the planner's experience and skills. The generalized benefits of auto‐contouring are decreased time required, improved quality, consistency, accuracy, and standardization.^13^, ^14^ RT in combination with AI as a proposed tool for auto‐contouring could potentially improve patient treatments.^15^

In this work, we present a novel solution based on partial OAR contouring that saves time and improves consistency for contours delineation and treatment planning. Partial OAR contouring is a technique where OARs are contoured only at a specified distance to a patient's planning target volume (PTV). In this study, we evaluated the efficacy of the partial uninvolved OAR technique using custom KBP models. To then refine and enhance the process we introduced AI. We created a separate plan utilizing AI contouring with our KBP model based on partial OARs uninvolved and contrasted it with the plans generated using the KBP model relying on manual full contours and a KBP model relying on manual partial uninvolved OARs.

## 2 METHODS AND MATERIALS

### 2.1 Patient selection

Sixty prostate patients with a prescription of 70 Gy/28Fx were chosen at random from a database of delivered treated plans to be retrospectively studied. They were anonymized and divided into two groups, 30 patients with a SpaceOAR, and 30 patients without SpaceOAR. Groups were created to see any potential advantages or disadvantages of our model in the presence of SpaceOAR. A SpaceOAR is an injectable rectal spacer made of polyethylene glycol (PEG).^16^

### 2.2 Partial uninvolved contours

**FIGURE 1:**
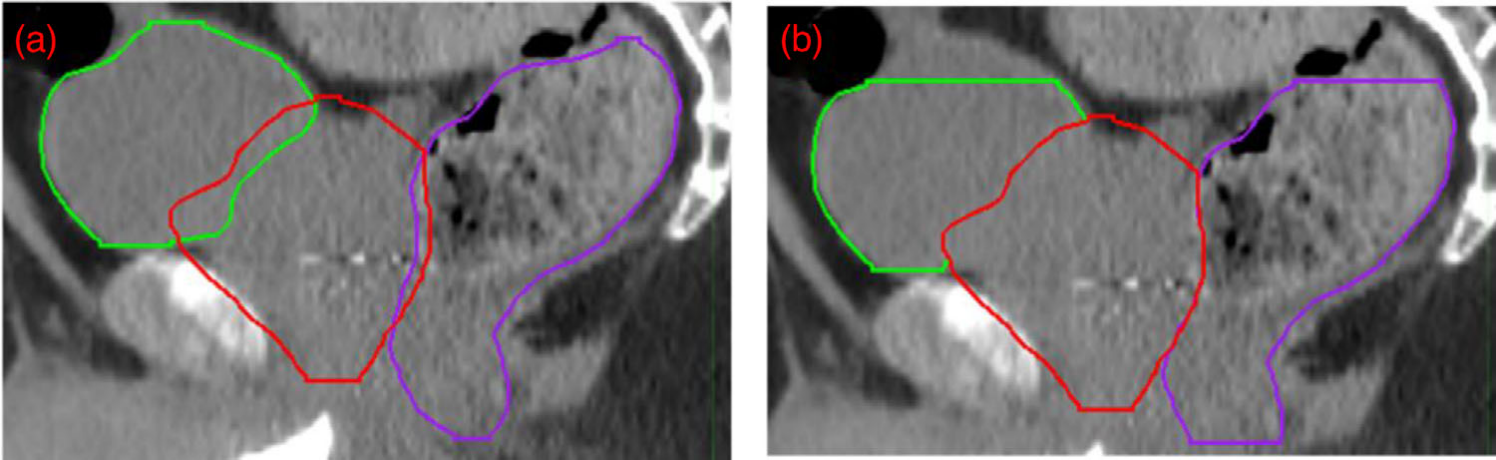
(a) Full planning target volume (PTV), bladder, and rectum contours. (b) Partial uninvolved bladder and rectum contours, that is, contours that are within 1 cm of the PTV in the superior–inferior dimension and do not overlap with the PTV.

The partial uninvolved OAR contours are constructed such that only OAR volumes on CT slices within 1 cm distance to the PTV edge superiorly and inferiorly are considered. The overlapping region with PTV is then removed in all dimensions. This was the case for both the bladder and rectum (Figure 1).

### 2.3 KBP models and plans

We developed two KBP models, Rapid Plan (RP) and Rapid Plan partial uninvolved (RP_Part_Un), for this study (Varian Medical Systems, Palo Alto, CA, USA). RP, a commercially available version of KBP that has been described in previous works,^17^, ^18^ was used. Seventy previous clinically approved volumetric modulated arc therapy (VMAT) plans each prescribed with 70 Gy/28 fractions were used to develop the models. From these plans, full organ contours were defined for the RP model; while partial uninvolved contours were defined for the RP_Part_Un model. Using these models, three plans were then created and compared for each validation patient. All plans generated in this study have RP models based on PTV, rectum, bladder, and femoral heads, and all these structures were using for plan optimization. RP_Part_Un model and plan use partial uninvolved rectum and bladder structures. Plan 1 was named RP plan, which used the RP model and fully contoured structure set. Plan 2 was named RP_Part_Un plan, which used the RP_Part_Un model and partial uninvolved contour set. For further analysis, we implemented AI to see if any additional benefits were provided. MIM Contour ProtegeAI (MIM Maestro, Cleveland, OH) was used to perform AI‐based segmentation.^19^ Plan 3 was named MIM AI_Part_Un which used the RP_Part_Un model; however, AI segmentation was used to create the partial contours, unlike plan 2.

### 2.4 Plan evaluation

For determining OAR (bladder and rectum) sparing, *D*_max_ and *D*_mean_ along with the percent volume receiving a dose over a range (*V*_10 Gy_–*V*_70_ _Gy_) were compared. Percent volume receiving 40 Gy (*V*_40_ _Gy_) was analyzed for both left and right femoral heads. These values were compared for the fully contoured plan (RP) against the partially contoured plan (RP_Part_Un). To ensure fairness in all dosimetric comparisons, entire OAR contours were used for all comparisons instead of partial contours. All plans were normalized so that 100% of the prescribed dose covered 98% of the PTV.

The MIM AI_Part_Un plan was subsequently compared to the RP_Part_Un plan for the select dosimetric indices mentioned previously. Geometric evaluations, dice similarity coefficient (DSC), and overlay index (OI) between the OAR contours from partial‐contoured manual structure sets and partial‐contoured AI structure sets were analyzed. Also, geometric evaluations for fully contoured manual structure sets and fully contoured AI structure sets were performed. Calculations for descriptive statistics included the determination of mean values and standard deviations. Student's *t*‐tests were done for quantitative comparisons, and two‐sided *p*‐values ≤ 0.05 were considered statistically significant.

## 3 RESULTS

As we studied the use of AI for contouring across both patient groups, we observed an increase in the similarity and accuracy of AI to the manual contours for the partial contours versus the full manual OAR contours (see Table 1). When comparing the DSC and OI for full contours to the partial contours, in patients with SpaceOAR, all comparisons showed significantly improved agreement for both organs. The same results were observed for patients without SpaceOAR. This indicates that there was a higher degree of concordance between the contours when only partial contours were considered.

**TABLE 1:** Geometric comparison between the full and partial manual and MIM AI contours.

	Full contours		Partial contours	
	DSC	OI	DSC	OI
Bladder				
SpaceOAR	0.92 ± 0.05	0.92 ± 0.05	0.96 ± 0.03	0.94 ± 0.06
No SpaceOAR	0.94 ± 0.02	0.95 ± 0.04	0.96 ± 0.02	0.96 ± 0.04
				
Rectum				
SpaceOAR	0.81 ± 0.05	0.78 ± 0.07	0.85 ± 0.04	0.85 ± 0.06
No SpaceOAR	0.79 ± 0.10	0.72 ± 0.14	0.86 ± 0.08	0.82 ± 0.11

AI, artificial intelligence; DSC, dice similarity coefficient; OAR, organ‐at‐risk; OI, overlay index.

### 3.1 Comparison between RP and RP_Part_Un

For patient groups with SpaceOAR, the *D*_max_ and *D*_mean_ of the bladder for RP_Part_Un plans, statistically, were significantly smaller than those for the RP plan (*p* ≤ 0.05), and no statistically different results were observed for *D*_max_ and *D*_mean_ for the rectum. For patient groups without SpaceOAR, there was a significant improvement on rectum *D*_mean,_ and no differences were observed for rectum *D*_max_ as well as bladder *D*_max_ and *D*_mean_. All values are shown in Table 2.

**TABLE 2:** Average doses for rectum *D*_max_, rectum *D*_mean_, bladder *D*_max_, and bladder *D*_mean_ for RP, RP_Part_Un, and MIM AI_Part_Un.

	SpaceOAR			No SpaceOAR		
	RP	RP_Part_Un	MIM AI_Part_Un	RP	RP_Part_Un	MIM AI_Part_Un
Rectum *D*_max_ (Gy)	64.82 ± 9.13	65.53 ± 8.42	64.45 ± 9.12**	72.64 ± 5.77	74.72 ± 1.10	72.98 ± 2.69**
Rectum *D*_mean_ (Gy)	17.16 ± 3.84	16.95 ± 3.49	17.18 ± 3.36	24.11 ± 4.70	22.24 ± 4.56*	21.84 ± 4.85
Bladder *D*_max_ (Gy)	73.04 ± 0.48	72.85 ± 0.44*	72.92 ± 0.45	69.96 ± 16.40	72.77 ± 13.33	74.95 ± 1.07
Bladder *D*_mean_ (Gy)	28.26 ± 8.29	27.21 ± 8.12*	27.42 ± 8.23**	20.85 ± 9.29	22.03 ± 8.13	23.21 ± 7.25

* Indicates *p* ≤ 0.05 for RP and RP_Part_Un comparison.

** Indicates *p* ≤ 0.05 for RP_Part_Un and MIM AI_Part_Un comparison. AI, artificial intelligence; OAR, organ‐at‐risk; RP, Rapid Plan; RP_Part_Un, Rapid Plan partial uninvolved.

For the patient sample used in this study, the doses to both left and right femoral heads were less of a concern in the treatment planning process. As an example, the average volumes receiving 40 Gy (V40 Gy) are listed in Table 3 for both left and right femoral head. No significant differences (within 1%) were observed among all three plans for both SpaceOAR and non‐SpaceOAR patient groups.

**TABLE 3:** Comparisons of *V*_40_ _Gy_ for left and right femur heads.

	SpaceOAR			No SpaceOAR		
	RP	RP_Part_Un	MIM AI_Part_Un	RP	RP_Part_Un	MIM AI_Part_Un
Left femur	0.02 ± 0.10	0.19 ± 0.77	0.12 ± 0.32	1.56 ± 5.24	1.23 ± 4.37	0.16 ± 0.53
Right femur	0.02 ± 0.07	0.39 ± 0.98	0.49 ± 1.36	1.32 ± 6.88	0.29 ± 0.80	0.9 ± 1.97

AI, artificial intelligence; OAR, organ‐at‐risk; RP, Rapid Plan; RP_Part_Un, Rapid Plan partial uninvolved.

**FIGURE 2:**
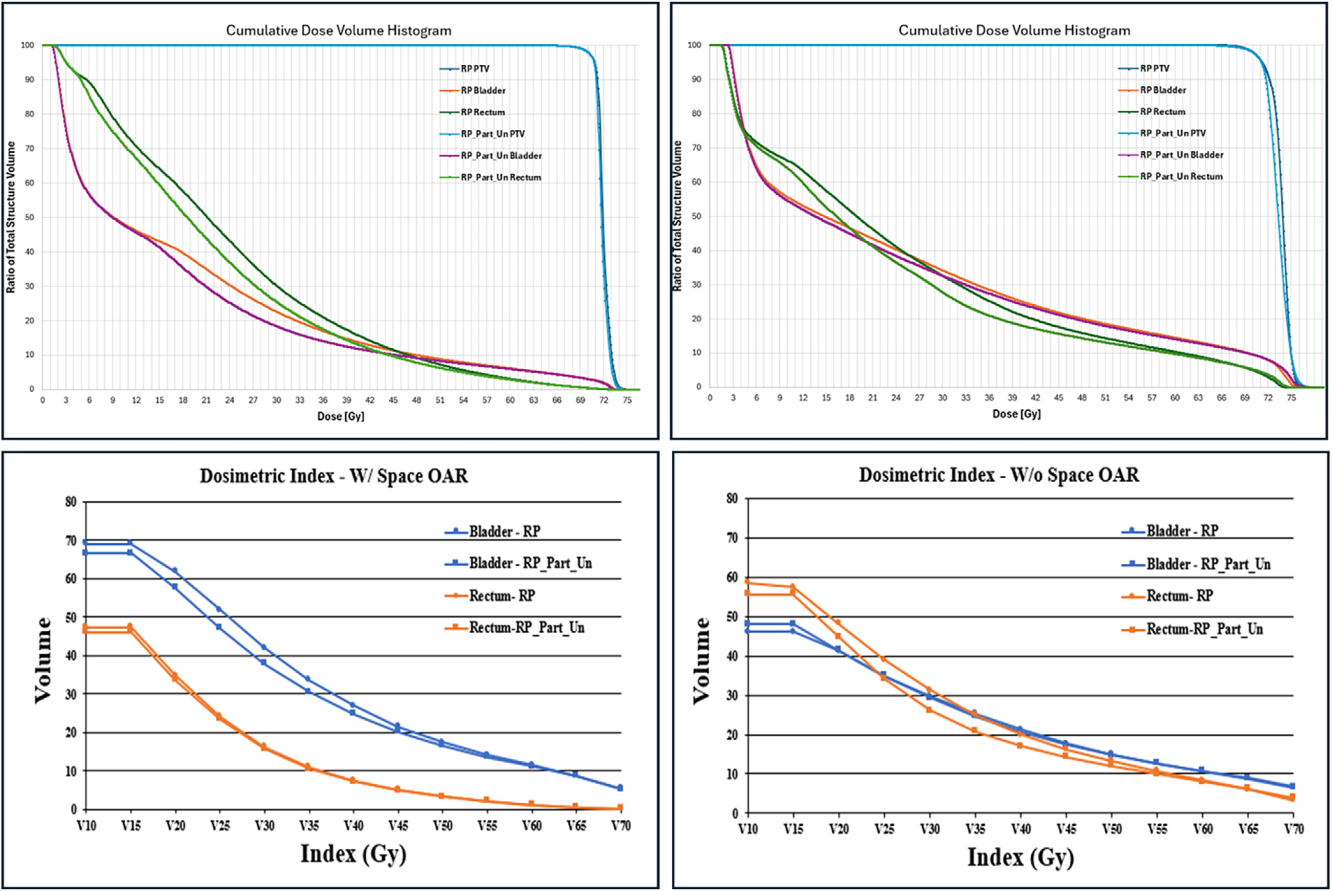
Comparison of RP and RP_Part_Un plans. Two representative patient DVHs with (top left) and without (top right) SpaceOAR and dosimetric comparisons (*V*_10_–*V*_70_) for space (bottom left) and no space (bottom right) OAR. DVH, dose‐volume histogram; OAR, organ‐at‐risk; RP, Rapid Plan; RP_Part_Un, Rapid Plan partial uninvolved.

As shown in Figure 2, for two representative patients over the percent volume range (*V*_10 Gy_–*V*_70 Gy_) compared to RP plans, the RP_Part_Un showed improved bladder dosimetry for the patient group with SpaceOAR, and improved rectum dosimetry for the patient group without SpaceOAR.

### 3.2 Comparison between RP_Part_Un and MIM_Part_Un

For patient groups with SpaceOAR, the *D*_max_ of the rectum was significantly lower for MIM AI_Part_Un plan. The *D*_mean_ of the bladder was also significantly different; however, the RP_Part_Un plan was smaller. No statistically significant results were observed for bladder *D*_max_ and rectum *D*_mean_. For patient groups without SpaceOAR, the *D*_max_ of rectum was observed to be significantly lower for MIM AI_Part_Un. No statistically significant results were observed for rectum *D*_mean_ as well as bladder *D*_max_ and *D*_mean_. All values are shown in Table 2.

**FIGURE 3:**
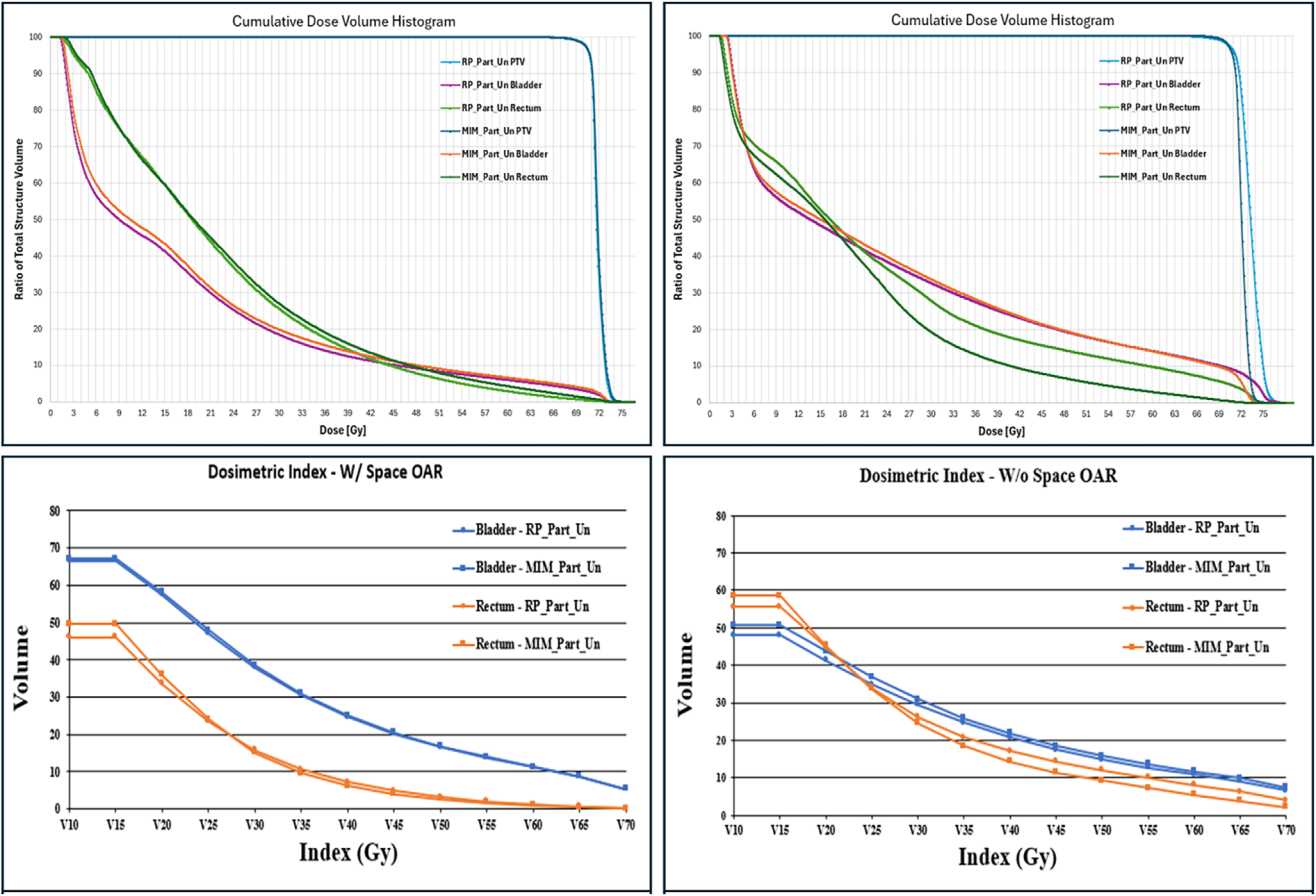
Comparison of RP_Part_Un and MIM AI_Part_Un plans. Two representative patient DVHs with (top left) and without (top right) SpaceOAR and dosimetric comparisons (*V*_10_–*V*_70_) for space (bottom left) and no space (bottom right) OAR. DVH, dose‐volume histogram; OAR, organ‐at‐risk; RP_Part_Un, Rapid Plan partial uninvolved.

As shown in Figure 3, for two representative patients over the percent volume range (*V*_10_–*V*_70_) starting around *V*_30_ _Gy_, compared to RP_Part_Un plans the MIM AI_Part_Un gave improved rectum dosimetry for both patient groups.

The ratios of RP_Part_Un and RP, MIM AI_Part_Un and RP_Part_Un, for *D*_max_, *D*_mean_, *V*_70_ _Gy_, and *V*_65_ _Gy_ are shown in Table 4 for the OARs (bladder and rectum).

**TABLE 4:** Parity comparison of dosimetric values obtained from plans. *D*_max_, *D*_mean_, *V*_70_ _Gy_, and *V*_65_ _Gy_ were compared.

OAR/plans comparison		*D*_max_	*p*‐value	*D*_mean_	*p*‐value	*V*_70 Gy_	*p*‐value	*V*_65 Gy_	*p*‐value
Rectum ratio (RP_Part_Un/RP)	w/o space	1.03	0.06	0.92	***0.02***	1.21	***<0.01***	1.01	0.87
	space	1.01	0.07	0.99	0.38	1.14	***<0.01***	1.04	***<0.01***
Rectum ratio (MIM AI_Part_Un /RP_Part_Un)	w/o space	0.98	***<0.01***	0.98	0.55	0.55	***<0.01***	0.62	***<0.01***
	space	0.98	***0.02***	1.01	0.22	0.93	0.29	0.87	0.08
Bladder ratio (RP_Part_Un/RP)	w/o space	1.04	0.48	1.06	0.50	1.02	0.83	1.02	0.85
	space	1.00	***<0.01***	0.96	***<0.01***	0.98	***0.01***	0.98	***0.02***
Bladder ratio (MIM AI_Part_Un/RP_Part_Un)	w/o space	1.03	0.38	1.05	0.12	1.12	0.06	1.10	0.08
	space	1.00	0.31	1.01	***0.03***	1.02	***<0.01***	1.01	***<0.01***

A value <1 indicates a better performance by the former, 1 indicates parity, and >1 indicates a better performance by the latter in each comparison. A *p* ≤ 0.05 were bolded and italicized for emphasis. AI, artificial intelligence; OAR, organ‐at‐risk; RP, Rapid Plan; RP_Part_Un, Rapid Plan partial uninvolved.

## 4 DISCUSSION

In our retrospective study, patients were separated into two groups, individuals with and without a SpaceOAR. The purpose of a SpaceOAR insertion into a patient is to create a physical separation of the rectum to the prostate, allowing for dose reduction to the rectum. Since the rectum in the SpaceOAR patients is typically displaced from the PTV compared to non‐SpaceOAR patients, these patients will have less overlap between the PTV and rectum, and so the use of partial contours should have less impact on plan quality. We observed for the SpaceOAR patients, no significant difference in *D*_mean_ and *D*_max_ for the rectum when between the RP and RP_Part_Un plans. Which is why little to no additional benefits were observed.

For patient groups with SpaceOAR, RP_Part_Un plans gave the *D*_max_ and *D*_mean_ values significantly smaller compared to RP plan for the bladder, and no significant dose difference observed for the rectum. For patient groups without SpaceOAR, compared to RP plans, we observed a significant improvement on rectum *D*_mean_, and no differences noticed for bladder *D*_max_ and *D*_mean_ for RP_Part_Un plans. For non‐SpaceOAR patient groups, the rectum is the closest OAR to the target and typically requires optimizer effort most in the planning process. Our study showed the partial uninvolved OAR volumes may give us superior dosimetry to the rectum in our patient sample. Similarly for patient group with SpaceOAR, less optimization effort will be spent on the rectum compared to the bladder, this is why we observed an improved bladder dosimetry from the partial uninvolved contours.

Previous works have been focused on using different geometric indices to quantify AI contour accuracy with varied results. However, the important matrix has been overlooked is the plan quality generated based on those AI contours. This work shows that MIM AI contours can be used to create high quality treatment plan. When the MIM AI_Part_Un plan was used for further comparison to the RP_Part_Un plan, the max dose for rectum was significantly lowered for both patient groups. A possible rationale for why a difference was seen in rectum *D*_mean_ and *D*_max_ but not bladder could be assumed from the geometric indices of the rectum. Rectum typically has a lower DSC and OI.^6^, ^20^, ^21^ Although we obtained higher indices when considering only the partially uninvolved volumes, the values for the rectum are lower and therefore have more of a possibility for variation in dose calculations. Due to its simplicity, this automated model could potentially be used in various other clinics.

We investigated reproducibility and the auto‐segmentation success via measuring DSC and OI. We observed that as we incorporated partial contours instead of the full contours the DSC and OI values increased (Table 1). The higher DSC and OI values that were observed can be attributed to the reduced range and limited CT images utilized for the delineation of the OAR contours. Inter‐observer variability has been shown in previous studies by organ volume measurement.^22^ Using the partial contour technique, the variation in volume for the OARs can be decreased. In theory, the more volume an expert observer has to contour, the more possibility for error and variation. So, a reduction of the volume to be contoured would reduce the likelihood of error and variation. When AI was introduced, it was shown to produce a more similar contour set with the manual plan when using the partially uninvolved model. This agrees with our theory that consistency can be improved by using partial contours. The degree of variation is reduced as the *DSC* and *OI* values increase as shown in Table 1. This is important in treatment planning because poor delineation can lead to toxicity to these normal tissues.

To compare how similar the dosimetric values for the OARs were between the plans, a ratio was done. In Table 4, in general, *D*_max_, *D*_mean_, *V*_70_ _Gy_, and *V*_65_ _Gy_ were near the value of 1. This indicates that MIM AI_Part_Un and RP_Part_Un plans resulted in similar dosimetric values. The lowest values (0.55, 0.62) were observed when comparing the ratio of MIM AI_Part_Un to RP_Part_Un for average *V*_70_ _Gy_ and *V*_65_ _Gy_, respectively, for rectum with no SpaceOAR patients. As we see in Figure 3, for the no SpaceOAR patients, at *V*_70_ _Gy_ and *V*_65_ _Gy_ we see a significant dose difference for MIM AI_Part_Un compared to RP_Part_Un. MIM AI_Part_Un calculated dose is nearly at zero while RP_Part_Un is nearly double that. This explains why the ratio was nearly half for *V*_70_ _Gy_ and *V*_65 Gy._

A possible limitation of this study is a quantitative measurement in which the plans could affect treatment planning time. Our assumption is that we expect using partial contours would result in time saving. As for AI segmentation, from plenty of previous studies, treatment planning time is improved with implementing AI contouring.^15^, ^16^, ^23^, ^24^, ^25^, ^26^ Another limitation is the application of this model on different OARs and other disease sites.

Our study illustrates a similar concept in which planners use clipped structures for plan optimization. Overlapping regions of OARs and PTV typically receive high dose and distanced regions receive low to no dose. The results demonstrate that focusing on contouring only OARs that are partial and within a specified range from the PTV has the potential to decrease treatment planning time, enhance consistency, minimize resource utilization, and achieve comparable doses for the PTV and OARs.

## 5 CONCLUSION

This work shows that the plan created with uninvolved partial contours to only a specified distance (1 cm) from the PTV provides comparable dosimetry to the plan created with fully contours. To further study the benefits of using uninvolved partial contours, an auto‐segmentation model was then used. The auto‐segmented model provides an equivalent result. This would additionally decrease treatment planning time and improve consistency. These results from this study would suggest AI partial contours can be implemented for treatment planning for prostate patients.

## AUTHOR CONTRIBUTIONS

Ositomiwa O. Osipitan collected the data, performed the analysis, and drafted the manuscript. David Wiant assisted with the study design and drafted the manuscript. Han Liu designed the study, assisted with the data analysis, and drafted the manuscript.

## CONFLICT OF INTEREST STATEMENT

This research was supported by a grant from Varian Medical Systems, Palo Alto, CA.

## Data Availability

Authors are not able to share data at this time.
